# Sequential Oxygenation Index and Organ Dysfunction Assessment within the First 3 Days of Mechanical Ventilation Predict the Outcome of Adult Patients with Severe Acute Respiratory Failure

**DOI:** 10.1155/2013/413216

**Published:** 2013-02-18

**Authors:** Hsu-Ching Kao, Ting-Yu Lai, Heui-Ling Hung, Yu-Mu Chen, Po-An Chou, Chin-Chou Wang, Meng-Chih Lin, Wen-Feng Fang

**Affiliations:** ^1^Division of Pulmonary and Critical Care Medicine, Department of Internal Medicine, Kaohsiung Chang Gung Memorial Hospital, Chang Gung University College of Medicine, Niaosung, Kaohsiung 833, Taiwan; ^2^Department of Respiratory Therapy, Kaohsiung Chang Gung Memorial Hospital, Chang Gung University College of Medicine, Kaohsiung 833, Taiwan; ^3^Department of Respiratory Care, Chang Gung University of Science and Technology, Chiayi 813, Taiwan

## Abstract

*Objective*. To determine early predictors of outcomes of adult patients with severe acute respiratory failure. *Method*. 100 consecutive adult patients with severe acute respiratory failure were evaluated in this retrospective study. Data including comorbidities, Sequential Organ Failure Assessment (SOFA) score, Acute Physiological Assessment and Chronic Health Evaluation II (APACHE II) score, PaO_2_, FiO_2_, PaO_2_/FiO_2_, PEEP, mean airway pressure (mPaw), and oxygenation index (OI) on the 1st and the 3rd day of mechanical ventilation, and change in OI within 3 days were recorded. Primary outcome was hospital mortality; secondary outcome measure was ventilator weaning failure. *Results*. 38 out of 100 (38%) patients died within the study period. 48 patients (48%) failed to wean from ventilator. Multivariate analysis showed day 3 OI (*P* = 0.004) and SOFA (*P* = 0.02) score were independent predictors of hospital mortality. Preexisting cerebrovascular accident (CVA) (*P* = 0.002) was the predictor of weaning failure. Results from Kaplan-Meier method demonstrated that higher day 3 OI was associated with shorter survival time (log-Rank test, *P* < 0.001). *Conclusion*. Early OI (within 3 days) and SOFA score were predictors of mortality in severe acute respiratory failure. In the future, prospective studies measuring serial OIs in a larger scale of study cohort is required to further consolidate our findings.

## 1. Introduction

Acute respiratory failure is one of the most common causes for the emergency admission of patients to intensive care units [1]. Epidemiological studies have estimated the annual incidence of acute respiratory failure in the United States (US) to be between 77.6 and 430 patients per 100,000 [1–4]. The mortality rates of acute respiratory failure in western countries ranged from 41% to 44% [3–5] and rise with the increase in severity and number of organ dysfunction [6]. Although in the past decades, clinicians have had better understanding of the physiological and biochemical effects of mechanical ventilation on the lungs and adapted various ventilation strategies that improved survival of patients with acute respiratory failure, this condition remains a major source of morbidity and mortality in the modern world [7].

The medical expenditure on treatment of critically ill patients with acute respiratory failure is substantial. The estimated health care costs related to critical care are approximately 0.7 percent of the annual gross domestic product in US [8, 9]. The financial costs and human resources required for treating critically ill patients are expected to rise with the aging population. For this reason, any modality utilized in improving quality of care on critically ill patients is crucial. Early prediction of outcome of patients with acute respiratory failure enables prompted adjustment of treatment strategy and more efficient allocation of medical resources.

Traditionally, a number of severity scoring systems such as Acute Physiology and Chronic Health Evaluation (APACHE) score, Simplified Acute Physiologic Score (SAPS), Mortality Prediction Model (MPM), and Sequential Organ Failure Assessment (SOFA) score are frequently adopted for severity evaluation and as outcome predictors in ICU patients [10]. Despite their widespread use, limitation such as lead time bias [11] and effects of constantly evolving medical technology in modern ICU setting [12] require the clinicians to update and reevaluate the usefulness of these scoring systems. Moreover, some of the scoring systems require a variety of laboratory data and physiologic parameters for equation calculation and thus bear inherent complexity to use. Furthermore, no scoring system has incorporated the effect of ventilator parameters, such as positive end-expiratory pressure (PEEP) and mean airway pressure (mPaw), on patient outcome. Thus the aim of this study is to investigate, besides existing severity scoring systems, any early predictors of outcome of patients with severe acute respiratory failure and to reevaluate the predictability of the severity scoring system routinely used in our ICU.

## 2. Patients and Methods

### 2.1. Study Design, Setting, and Patients

This retrospective study was conducted at one of the medical intensive care units (ICU) in Kaohsiung Chang Gung Memorial Hospital, a 2,400-bed, tertiary teaching hospital in southern Taiwan. The study was conducted from January 1, 2010, to July 31, 2010, for adult patients (aged 18 years or more) admitted consecutively to the designated medical ICU who fulfilled criteria for severe acute respiratory failure. Severe acute respiratory failure was defined as acute respiratory insufficiency requiring ventilator support for at least 24 hours. The study was approved by the Institutional Review Board of Chang Gung Memorial Hospital, and the requirement for patient consent was waived.

### 2.2. Data Collection

Clinical data retrieved from medical records included age, gender, initial admitting diagnosis, source of admission, hospital duration, previous ICU admission within 2 years, underlying comorbidities, tracheostomy status prior to current admission, Sequential Organ Failure Assessment (SOFA) score on the day of admission, and Acute Physiological Assessment and Chronic Health Evaluation II (APACHE II) score. Respiratory and ventilator parameters retrieved included PaO_2_, FiO_2_, PEEP, and mPaw on the 1st and the 3rd day of mechanical ventilation. Both oxygenation index (OI) and PaO_2_/FiO_2_ ratio on the 1st and 3rd day of ICU admission were calculated and recorded, respectively, for each patient. OI was defined as 100 × mPaw/(PaO_2_/FIO_2_). The change in OI values from the 1st day to the 3rd day was obtained by subtracting day 1 OI from day 3 OI. The primary outcome was overall hospital mortality. Secondary outcome measure was ventilator weaning failure, which was defined as the failure of complete autonomy from the ventilator for more than 5 days.

### 2.3. Statistical Analysis

Categorical variables were analyzed using the chi-square test or Fisher's exact test where appropriate, and continuous variables were compared using Student's *t*-test or the Mann-Whitney *U* test. Multivariate logistic regression analysis was performed to identify risk factors for hospital mortality and weaning success. All variables considered as risk factors with a *P* value <0.10 in univariate analysis were entered into the multivariate model. If individual variables associated with mortality had a *P* value <0.05 in the multivariate model, a backward elimination procedure was used to identify the final independent risk factors. The discriminating power of different independent risk factors were determined using the receiver operating characteristic (ROC) curves, and areas under the curve were compared. The cutoff value of each risk factor for mortality among ventilated ICU patients was analyzed according to ROC curves. Kaplan-Meier method was used to demonstrate the relevance among four stratified oxygenation index groups (stratified as values <5, 6~10, 11~15, and >15) and mortality. Results are presented as absolute numbers (percentage) or mean ± standard deviation (SD). Adjusted odds ratios (AORs) and 95% confidence intervals (CIs) were reported for logistic regression analysis. A two-tailed *P* value of <0.05 was considered significant. All statistical analysis was performed using the SPSS 19.0 software package (SPSS Inc., Chicago, IL, USA).

## 3. Results

### 3.1. Patient's Characteristics

A total of 145 consecutive patients were admitted into the designated MICU within a 7-month period (January 1, 2010 to July 31, 2010), of which 34 patients did not fulfill the criteria of severe acute respiratory failure. Of the 111 patients who met the criteria for severe acute respiratory failure and received mechanical ventilation, 11 had critical data that were either incomplete or missing and were not entered into the statistical analysis. The remaining 100 patient's baseline characteristics and ventilator parameters on the 1st and 3rd day were shown in Table 1. Patients' mean age was 72.1(15.8) years, 58% were males. The main source of patient's admission were from the emergency department (55%) and hospital wards (38%). 30 (30%) patients had previous ICU admission history. Overall hospital mortality was 38%. The most common comorbidities (Table 1) were hypertension 62(62%), followed by diabetes mellitus 35(35%). The mean value of patients' APACHE II score was 25.82(6.74), while their SOFA score on the 1st day of admission was 4.71(2.71). The mean hospital duration was 38.1(30.5) days. Etiologies leading to respiratory failure requiring ICU admission were summarized in Table 2. The most common cause was community-acquired pneumonia (30%), followed by urosepsis (13%).

### 3.2. Hospital Mortality

Table 3 shows results of bivariate analysis of baseline characteristics of survivors and nonsurvivors. High initial SOFA score was found correlated with mortality (*P* = 0.007). No significant difference was found between the 2 groups in age (*P* = 0.096), gender (*P* = 0.99), source of admission (*P* = 0.48), previous ICU admission (*P* = 0.47), APACHE II score (*P* = 0.15), and background comorbidities.  Table 4 shows respiratory and ventilator parameters of both patient groups. High day 1 (*P* = 0.002) and day 3 FiO_2_ (*P* = 0.001), high day 3 Paw (*P* < 0.001), low day 3 PaO_2_/FiO_2_ (*P* = 0.01), high day 3 OI (*P* < 0.001), and increasing OI from day 1 to day 3 (*P* < 0.001) were found significantly correlated with mortality. In contrast, day 1 and day 3 PaO_2_ (*P* = 0.14 and *P* = 0.07, resp.) day 1 and day 3 PEEP (*P* = 0.38 and *P* = 0.42, resp.) day 1 mPaw (*P* = 0.69), day 1 PaO_2_/FiO_2_ (*P* = 0.87,) and day 1 OI (*P* = 0.33) were not statistically correlated with mortality.

### 3.3. Predictors of Overall Hospital Mortality

Using univariate analysis of factors capable of predicting overall hospital mortality, nonsurvivors were found to have a higher initial SOFA score (AOR 1.24, 95% confidence interval (CI) 1.05–1.47, *P* = 0.01), a higher day 3 mPaw (AOR 1.36, 95% CI 1.14–1.63, *P* = 0.001,) a lower day 3 P/F (AOR 0.99, 95% CI 1.11–1.44, *P* = 0.006,) a higher day 3 OI (AOR 1.26, 95% CI 1.11–1.44, *P* < 0.001,) and increasing OI from day 1 to day 3 (AOR 1.12, 95% CI 1.03–1.22, *P* = 0.008). Independent risk factors for hospital mortality were identified using multivariate logistic regression analysis (Table 5), factors identified include high initial SOFA score on admission (AOR 1.27, 95% CI 1.04–1.55, *P* = 0.02,) and high day 3 OI values (AOR  =  1.49, 95% CI 1.13–1.95, *P* = 0.004).

### 3.4. Receiver Operating Characteristic Curves

**Figure 1:**
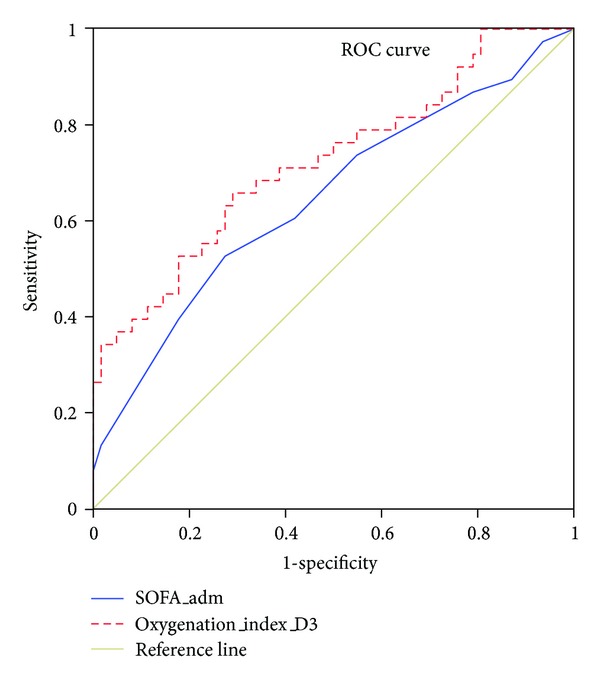
ROC curve analysis for predictability of hospital mortality between Day 3 OI and SOFA score.

ROC curves were plotted to identify cutoff values that would best determine hospital mortality of ICU patients (Figure 1). The optimal cutoff values for SOFA score and day 3 OI were 5.5 and 3.79, respectively. These values yielded a sensitivity and specificity of 53% and 73%, for the SOFA score, 69% and 71% for day 3 OI, respectively. The area under the ROC curve indicated higher sensitivity and specificity for the day 3 OI than SOFA score for determining hospital mortality (0.72 versus 0.65, *P* < 0.001).  Table 6 shows comparison of cutoff values, sensitivity, specificity, AUC, and *P* value for these two physiologic scores.

### 3.5. Relevance of Day 3 OI Stratified Values and Survival Time

**Figure 2:**
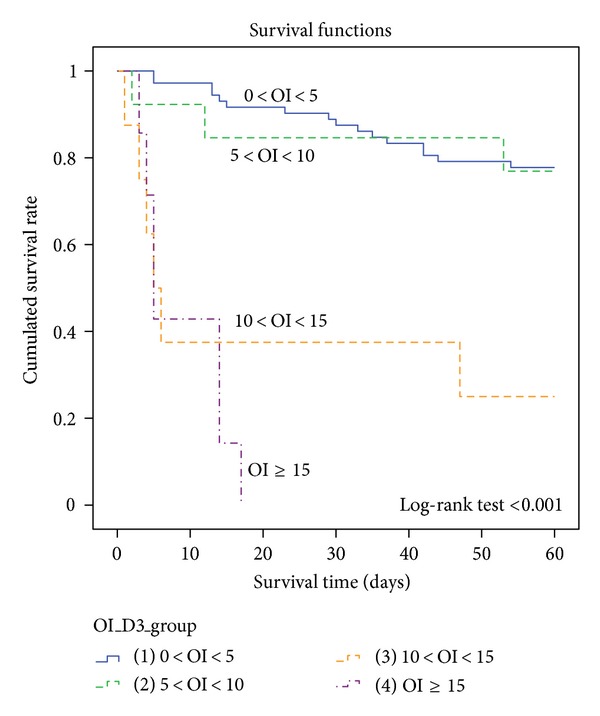
Kaplain Meier curve of stratified day 3 OI groups versus survival time.

To illustrate the correlation between oxygenation index and survival time, we stratified day 3 oxygenation index into 4 groups (0  <  OI  <  5, 5  <  OI  <  10, 10  <  OI  <  15, and OI ≥ 15) using the Kaplan-Meier method. There was significant association between value of day 3 OI and the survival time as demonstrated by the log-rank test (*P* < 0.001) and the Kaplan-Meier plot (Figure 2). The plot demonstrated the group with the highest day 3 OI had the shortest survival time, while the group with the lowest day 3 OI has the longest survival time.

### 3.6. Weaning Outcomes

Table 7 shows baseline characteristics of patients successfully weaned from ventilator and those who failed to wean.  Table 8 shows respiratory and ventilator parameters of these patients on day 1 and day 3. Of the 62 survivors, 46 patients (74%) successfully weaned from ventilator, 16 patients (26%) failed to wean. Presence of preexisting cerebrovascular accident (CVA) (*P* < 0.001) and cancer history (*P* = 0.042) were associated with weaning failure. Although both groups had improving OI from day 1 to day 3, there was significant difference in the magnitude of OI change. The group who failed to wean had less OI improvement than the weaning success group (−1.06 versus −3.32, *P* = 0.008). However, there were no difference between the 2 groups in age (*P* = 0.08), gender (*P* = 0.86), all other background illnesses except CVA and cancer history, and all other ventilatory and respiratory parameters except OI change.

### 3.7. Predictors of Weaning Failure

Univariate analysis identified history of CVA as the potential risk factor for weaning failure (AOR  =  12.33, 95% CI 3.21–47.38, *P* < 0.001). OI change from day 1 to day 3 was also included in the multivariate analysis, since its association with weaning outcome is physiologically plausible and its *P* value is less than 0.1 in univariate logistic regression (AOR  =  0.792, 95% CI 0.62–1.02, *P* = 0.069). The results of multivariate analysis were shown in Table 9. Only history of CVA was identified as independent predictor of weaning failure (AOR  =  10.16, 95% CI 2.37–43.58, *P* = 0.002).

## 4. Discussion

This study has several findings. First, adults with severe acute respiratory failure receiving mechanical ventilation who did not survive were more likely to have initial high SOFA score, high FiO_2_, high day 3 mPaw, low day 3 PaO_2_/FiO_2_, high day 3 OI, and increasing OI from 1st to 3rd day of mechanical ventilation. When multivariate logistic regression analysis was implemented, SOFA score and 3rd day OI were found to be independent risk factors for hospital mortality, but the PaO_2_/FiO_2_ ratio was not. Second, OI had a better combined sensitivity and specificity than SOFA score in predicting mortality as reflected in our ROC analysis. Third, our study demonstrated significant correlation between the value of OI and survival time. When values of day 3 OI were stratified into 4 groups and plotted against survival time on the Kaplan-Meier graph, the group with highest OI value had a shortest survival time, while lower OI groups survived longer. Fourth, CVA was the only independent predictor of weaning failure from ventilator in our study.

OI was originally used in pediatric field as an index for prediction of mortality of infants with hypoxic respiratory failure [13, 14] and was also utilized as one of the clinical criteria for ECMO application (OI  >  40 on 2 or more blood gas measurements) [14–16]. In our study, we demonstrated OI measured on the 3rd day of mechanical ventilation predicts mortality better than the 1st day OI. This result was consistent with the study by Trachsel et al. on acute hypoxic respiratory failure pediatric patients, in which they found from serial measurements of OI over time since intubation, multiple logistic analysis showed that initial measurements were not as predictive as those at 24 hours and thereafter [17]. Several reasons may explain this phenomenon. First, in the early course of respiratory failure, simple therapeutic interventions, such as chest percussion, airway suctioning, and recruitment maneuvers will give room for significant improvement in a portion of patients with high OI at presentation and good outcome. Second, although not routinely performed and its frequency not recorded in our study, therapeutic bronchoscopy performed early in the course of respiratory failure in some of our patients would also provide some benefit on oxygenation and subsequently reducing OI values. Third, in the ICU of our institution, besides attending physicians, respiratory therapists were directly involved with manipulation and adjustment of the ventilator setting. Although respiratory therapists always adjusted ventilator setting according to patients' condition, there was, inevitably, individual variation among different respiratory therapists' routine in setting the parameters of ventilator. For example, to adjust ventilator setting in different patients with the same condition, different respiratory therapists may set slightly higher or lower FiO_2_, PEEP, or other parameters, especially at the onset of mechanical ventilation when the patient's condition was still unstable and required more frequent adjustment of ventilator setting. Small change in each parameter may have significant effect on the final mPaw, PaO_2_, and OI. However, to what degree this operator variation affected the final result was difficult to assess. In the future, serial OI measurements after intubation should provide more thorough understanding on how temporal difference in OI affecting mortality prediction. 

Numerous studies [18–20] demonstrated SOFA score predicts and correlates well with ICU mortality. For this reason it is widely used in the ICU setting, and this is again demonstrated in our study. However, our study showed day 3 OI has a higher sensitivity and specificity in predicting mortality than initial SOFA score. Due to lack of follow-up measurements of SOFA scores of our patients in the current study, it will be a premature conclusion to state that OI is a better predictor for mortality than SOFA score. To clarify this issue, a study comparing serial OI measurements with corresponding serial SOFA scores in predicting mortality of patients with respiratory failure will be required. Nevertheless, our result implies that besides SOFA score, APACHE II score, and other commonly used systemic scores, simple index such as OI could be useful in predicting mortality and should not be overlooked.

Our results demonstrated OI predicts mortality better than does PaO_2_/FiO_2_ in ventilated patients. Originally described in 1974 by Horovitz and colleagues, PaO_2_/FiO_2_ ratio was introduced in an attempt to overcome the limitations of alveolar-arterial (A-a) O_2_ pressure gradient and arterial alveolar (a-A) oxygen tension ratio (a/A ratio) and enable the evaluation of PaO_2_ at varying FiO_2_ [21]. PaO_2_/FiO_2_ is used in stratification of acute lung injury (ALI) and acute respiratory distress syndrome (ARDS) and is a commonly used respiratory index to describe the oxygenation status in the intensive care unit. Despite its widespread use, the relationship between PaO_2_/FiO_2_ and mortality has not been consistently demonstrated across several studies [22]. In fact, two problems arise when using PF as predictor of mortality. First, studies [23, 24] has shown that PEEP can significantly affect the value of the PaO_2_/FIO_2_ ratio. As a result, patients' classification among ARDS may change, mortality may thus be underestimated. Second, the predictive ability of the PaO_2_/FiO_2_ ratio on mortality [24] does not necessarily improve after adjusting PEEP. When comparing with PEEP, the mPaw may be a better indicator of lung recruitment. mPaw can be affected by any changes in PEEP, inspiratory-to-expiratory time ratio (I : E ratio), and tidal volume, OI is in turn altered by the change in mPaw. OI therefore reflects functional status of lung. For this reason, OI is more sensitive than the traditional PaO_2_/FiO_2_ ratio in assessing the oxygen exchanging status and severity of the lung injury and a better predictor of mortality.

In the past decade, low tidal volume ventilation (LTVV) has gained more popularity among clinicians as a measure to reduce mortality in ARDS. In patients with acute respiratory failure who do not meet the criteria but with risks for development of ARDS or ALI, evidence showed that LTVV with high PEEP may prevent development of ALI/ARDS [25]. Moreover, animal model showed early airway pressure release ventilation (APRV) can prevent ARDS in injured animals [26]. As discussed earlier, our study suggested OI is more sensitive than the traditional PaO_2_/FiO_2_ ratio in assessing the oxygen exchanging status. For this reason, OI can be a potential predictor for risks of development of ALI and ARDS in patients with acute respiratory failure and thus enables earlier modification of ventilation strategy.

Our study showed preexisting CVA was an independent predictor for ventilator weaning failure. Although acute stroke patients who require mechanical ventilation are known to carry poor outcomes [27, 28], preexisting cerebral infarction, and cerebral hemorrhage in patients admitted to ICU for a different disease are not necessarily associated with prolonged mechanical ventilation [29], which instead, may be related to the extent of neurological deficit. However, preexisting CVA was shown to be a risk factor for weaning failure in our study. This may be explained by the older age (78.3 year old versus 69.4 year old, *P* = 0.003) and higher number of comorbidities (2.9 versus 2.19, *P* = 0.01) in patients with preexisting CVA in our study.

Change in OI in the 1st 3 days was shown to correlate with weaning outcome in our study, though multivariate analysis failed to establish its role as an independent predictor. The relationship between OI and weaning outcome was discussed in several studies. In the study by Tseng et al., they demonstrated that congestive cardiac failure (*P* = 0.009), initial high oxygenation index value (*P* = 0.04), increased SOFA scores (*P* = 0.01), and increased APACHE II scores are independent predictors of ventilator dependence in patients with ventilator-associated pneumonia [30]. The study by Gajic et al. also suggested that age, OI, and cardiovascular failure three days after intubation are predictors of death or prolonged mechanical ventilation [31]. Comparing with the above mentioned studies, the heterogeneous nature of our ventilated patients may account for the different result in our study.

Our study has several limitations. First, a retrospective review of existing data was conducted, inevitably, disadvantage such as missing key data in small amount of patients would occur. This may reduce the representativeness of the sample. Second, the relatively small sample size implies a single data may have a greater influence on final results. Despite this, it did not affect our final conclusion or inference because our main results were highly significant since their *P* values were less than 0.05 or even less than 0.001. Third, it is not known which ventilator strategies (e.g., low tidal volume strategy, lung recruitment, etc.) were used in each patient and for how long, and if any additional therapies were used (e.g., inhalation medication or bronchoscopic intervention). However, as we had mentioned earlier that the ventilator strategy was decided by the attending physicians and the respiratory therapists, and such operator variation reflects the genuine condition in our ICU. Nevertheless, our study is the one of the few that, besides various clinical variables, has incorporated OI in the analysis of predictability on both mortality and weaning rate in adult patients, and compared it with the general severity scores.

## 5. Conclusion

This study suggested that elevated OI measured in the first 3 days of mechanical ventilation and high SOFA score are independent predictors of mortality in patients with acute respiratory failure requiring mechanical ventilation. OI is comparable with, if not superior than, general severity scores such as APACHE II and SOFA score in predicting mortality. Our study also suggested that by conducting serial OI measurements and monitoring trends over time may provide more useful information than any single measurement. In the future, prospective studies measuring serial OIs in a larger scale of study cohort will be required to further consolidate our findings.

## Figures and Tables

**Table 1:** Baseline characteristics of patients.

Characteristics	*N* = 100
Age (years)	72.14 (15.77)
Gender (*n* (%))	
Male	58 (58)
Female	42 (42)
Hospital duration (days)	38.1 (30.5)
Hospital mortality (*n* (%))	38 (38)
SOFA* score on admission	4.71 (2.71)
APACHE* II score	25.82 (6.74)
Source (*n* (%))	
Emergency department	55 (55)
Hospital ward	38 (38)
Other ICU* or other hospitals	7 (7)
Previous MICU* admission (*n* (%))	30 (30)
Comorbidities (*n* (%))	
Congestive heart failure	10 (10)
Hypertension	62 (62)
Chronic obstructive pulmonary disease	22 (22)
Pulmonary tuberculosis	9 (9)
CRF* with tracheostomy	11 (11)
Liver cirrhosis	10 (10)
Chronic kidney disease	19 (19)
Diabetes mellitus	35 (35)
Cerebrovascular accident	30 (30)
Hepatitis B	6 (6)
Hepatitis C	4 (4)
Cancer history	22 (22)
Respiratory and ventilator parameters	
FiO_2_* day1	0.58 (0.21)
PaO_2_* day 1 (mmHg)	140.46 (88.21)
PEEP* day 1 (cmH_2_O)	5.55 (2.63)
mPaw* day 1 (cmH_2_O)	11.99 (3.23)
PaO_2_/FiO_2_ day 1 (mmHg)	267.95 (181.11)
OI* day 1 (cmH_2_O/mmHg)	7.06 (6.01)
FiO_2_ day 3	0.43 (0.16)
PaO_2_ day 3 (mmHg)	109.79 (32.84)
PEEP day 3 (cmH_2_O)	5.8 (2.65)
mPaw day 3 (cmH_2_O)	11.46 (3.02)
PaO_2_/FiO_2_ day 3 (mmHg)	287.92 (116.3)
OI day 3 (cmH_2_O/mmHg)	5.74 (5.82)
OI change from day 1 to day 3	−1.31 (6.34)

*APACHE: Acute Physiological Assessment and Chronic Health Evaluation, CRF: chronic respiratory failure, FiO_2_: fraction inspired oxygen, ICU: intensive care unit, MICU: medical intensive care unit, mPaw: mean airway pressure, OI: oxygenation index, PaO_2_: arterial oxygen tension, PEEP: positive end expiratory pressure, SOFA: Sequential Organ Failure Assessment.

Variables are expressed as mean (standard deviation) and categorical data are expressed as number (percentage).

**Table 2:** Etiologies of severe acute respiratory failure.

Etiologies of respiratory failure (*n* (%))	
Acute myocardial infarction	1 (1)
Aortic aneurysm rupture	1 (1)
Community-acquired pneumonia	30 (30)
Hospital-acquired pneumonia	12 (12)
Healthcare-associated pneumonia	6 (6)
Chemical intoxication	1 (1)
Carbon monoxide poisoning	1 (1)
COPD* acute exacerbation	8 (8)
Acute stroke	2 (2)
Empyema	1 (1)
Organophosphate intoxication	1 (1)
Seizure disorder	1 (1)
Hyperosmolar hyperglycemic state	2 (2)
Infective endocarditis	1 (1)
Decompensated liver cirrhosis	1 (1)
Acute pulmonary embolism	1 (1)
Intra-abdominal infection	2 (2)
Sepsis unknown focus	4 (4)
Skin/soft tissue infection	3 (3)
Upper gastrointestinal bleeding	5 (5)
Tracheal stenosis	1 (1)
Urosepsis	13 (13)
Uremia	1 (1)
Ventilator-associated pneumonia	1 (1)

**Table 3:** Characteristics of survivors and nonsurvivors: bivariate analysis^#^.

Characteristics	Hospital mortality *N* = 38	Survivor *N* = 62	*P* values
Age (years)	68.37 (17.55)	74.45 (14.22)	0.1
Gender (*n* (%))			
Male	22 (57.9)	36 (58.1)	0.99
Female	16 (42.1)	26 (41.9)	
SOFA* score on admission	5.63 (3.07)	4.15 (2.3)	0.007
APACHE* II score	27.05 (7.44)	25.06 (6.21)	0.15
Source (*n* (%))			
Emergency department	18 (47)	37 (60)	
Hospital ward	17 (45)	21 (34)	0.48
Other ICU* or other hospitals	3 (8)	4 (6)	
Previous MICU* admission (*n* (%))	13 (42)	17 (27.4)	0.47
Comorbidities (*n* (%))			
Congestive heart failure	5 (13.2)	5 (8.1)	0.41
Hypertension	23 (60.5)	39 (62.9)	0.81
Chronic obstructive	7 (18.4)	15 (24.2)	0.5
pulmonary disease			
Pulmonary tuberculosis	2 (5.3)	7 (11.3)	0.31
CRF* with tracheostomy	3 (7.9)	8 (12.9)	0.44
Liver cirrhosis	6 (15.8)	4 (6.5)	0.13
Chronic kidney disease	7 (18.4)	12 (19.4)	0.91
Diabetes mellitus	12 (31.6)	23 (37.1)	0.57
Cerebrovascular accident	9 (23.7)	21 (33.9)	0.28
Hepatitis B	4 (10.5)	2 (3.2)	0.14
Hepatitis C	1 (2.6)	3 (4.8)	0.59
Cancer history	5 (13.2)	5 (8.1)	0.41

*APACHE: Acute Physiological Assessment and Chronic Health Evaluation, CRF: chronic respiratory failure, ICU: intensive care unit, MICU: medical intensive care unit, SOFA: Sequential Organ Failure Assessment.

^#^Continuous variables were analyzed by Student's *t*-test or Mann-Whitney *U *test, and categorical data by chi-square test.

Variables are expressed as mean (standard deviation) and categorical data are expressed as number (percentage).

**Table 4:** Respiratory and ventilator parameters of survivors and nonsurvivors on day 1 and day 3 of mechanical ventilation^#^.

Day 1 and day 3 Respiratory and ventilator parameters			
Characteristics	Hospital mortality *N* = 38	Survivor *N* = 62	*P* values
FiO_2_* day1	0.66 (0.21)	0.53 (0.19)	0.002
PaO_2_* day 1 (mmHg)	163.02 (104.78)	126.63 (73.87)	0.14
PEEP* day 1 (mmHg)	5.95 (3.94)	5.31 (1.28)	0.38
mPaw* day 1 (mmHg)	12.16 (3.35)	11.89 (3.18)	0.69
PaO_2_/FiO_2_ day 1 (mmHg)	271.84 (184.39)	265.56 (180.55)	0.87
OI* day 1	7.81 (7.62)	6.59 (4.78)	0.33

FiO_2_ day 3	0.51 (0.21)	0.38 (0.1)	0.001
PaO_2_ day 3 (mmHg)	102.72 (42.17)	114.12 (24.94)	0.07
PEEP day 3 (cmH_2_O)	6.11 (3.71)	5.58 (1.66)	0.42
mPaw day 3 (cmH_2_O)	12.89 (3.42)	10.58 (2.38)	<0.001
PaO_2_/FiO_2_ day 3 (mmHg)	245.84 (144.83)	313.7 (86.32)	0.01
OI day 3 (cmH_2_O/mmHg)	8.82 (8.16)	3.86 (2.26)	<0.001

OI change from day 1 to day 3 (cmH_2_O/mmHg)	1.0 (8.446)	−2.74 (4.08)	<0.001

*FiO_2_: fraction inspired oxygen, mPaw: mean airway pressure, OI: oxygenation index, PaO_2_: arterial oxygen tension, PEEP: positive end-expiratory pressure.

^#^Continuous variables were analyzed by Student's *t*-test or Mann-Whitney *U *test, and categorical data by chi-square test.

Variables are expressed as mean (standard deviation) and categorical data are expressed as number (percentage).

**Table 5:** Multivariate analysis of predictors of hospital mortality in patients with severe acute respiratory failure.

Variables	Odds ratio	CI	*P* values
SOFA* score	1.27	1.04–1.55	0.02
PaO_2_*/FiO_2_* day 3	1.01	1.00–1.02	0.05
OI* day 3	1.49	1.13–1.95	0.004
OI change from day 1 to day 3	1.11	0.99–1.24	0.07

*FiO_2_: fraction inspired oxygen, OI: oxygenation index, PaO_2_: arterial oxygen tension, SOFA: Sequential Organ Failure Assessment.

**Table 6:** Comparison of cutoff value, sensitivity, specificity, AUC*, and *P* values between OI and physiological severity scores.

Factor	Cutoff	Sensitivity	Specificity	AUC	*P* values
SOFA* score	5.5	53%	73%	0.647	0.01
Day 3 OI*	3.79	69%	71%	0.724	<0.001

*AUC: Area under curve, SOFA: Sequential Organ Failure Assessment, OI: oxygenation index.

**Table 7:** Characteristics of patients with different weaning outcomes: bivariate analysis^#^.

Characteristics	Weaning success *N* = 46	Weaning failure *N* = 16	*P* value
Age (years)	72.59 (15.04)	79.81 (10.14)	0.08
Gender (*n* (%))			
Male Female	27 (58.7) 19 (41.3%)	9 (56.3) 7 (43.8)	0.86
SOFA* score on admission	4.39 (2.30)	3.44 (2.22)	0.16
APACHE* II score	24.7 (6.62)	26.13 (6.43)	0.43
Source (*n* (%))			
Emergency department	27 (58.7)	10 (62.5)	
Hospital ward	17 (37.0)	4 (25)	0.42
Other ICU* or other hospitals	2 (4.3)	2 (12.5)	
Previous MICU admission (*n* (%))	10 (21.7)	7 (43.8)	0.09
Comorbidities (*n* (%))			
Congestive heart failure	5 (10.9)	0 (0)	0.17
Hypertension	28 (60.9)	11 (68.8)	0.57
Chronic obstructive	9 (19.6)	6 (37.5)	0.15
pulmonary disease			
Pulmonary tuberculosis	5 (10.9)	2 (12.5)	0.86
CRF* with tracheostomy	4 (8.7)	4 (25)	0.09
Liver cirrhosis	4 (8.7)	0 (0)	0.22
Chronic kidney disease	11 (23.9)	1 (6.3)	0.12
Diabetes mellitus	15 (32.6)	8 (50)	0.22
Cerebrovascular accident	9 (19.5)	12 (75)	<0.001
Hepatitis B	2 (4.3)	0 (0)	0.4
Hepatitis C	3 (6.5)	0 (0)	0.3
Cancer history	10 (21.7)	0 (0)	0.04

*APACHE: Acute Physiological Assessment and Chronic Health Evaluation, CRF: chronic respiratory failure, ICU: intensive care unit, MICU: medical intensive care unit, SOFA: Sequential Organ Failure Assessment.

^#^Continuous variables were analyzed by Student's *t*-test or Mann-Whitney *U *test, and categorical data by chi-square test.

Variables are expressed as mean (standard deviation) and categorical data are expressed as number (percentage).

**Table 8:** Day 1 and day 3 respiratory and ventilator parameters of patients with different weaning outcomes^#^.

Day 1 and Day 3 Respiratory and ventilator parameters			
Characteristics	Weaning success *N* = 46	Weaning failure *N* = 16	*P* value
FiO_2_* Day1	0.55 (0.2)	0.46 (0.17)	0.1
PaO_2_* Day 1 (mmHg)	129.11 (79.84)	119.5 (54.74)	0.69
PEEP* Day 1 (mmHg)	5.41 (1.33)	5.0 (1.10)	0.27
mPaw* Day 1 (mmHg)	12.0 (3.22)	11.56 (3.14)	0.64
PaO_2_/FiO_2_ Day 1 (mmHg)	259.02 (180.78)	284.36 (184.45)	0.63
OI* Day 1	6.99 (5.15)	5.46 (3.4)	0.27

FiO_2_ day 3	0.38 (0.08)	0.4 (0.14)	0.57
PaO_2_ Day 3 (mmHg)	114.63 (24.8)	112.64 (26.09)	0.79
PEEP Day 3 (cmH_2_O)	5.41 (1.33)	5.0 (1.1)	0.43
mPaw day 3 (cmH_2_O)	10.37 (2.44)	11.19 (2.14)	0.24
PaO_2_/FiO_2_ Day 3 (mmHg)	313.94 (76.81)	313.02 (112.25)	0.97
OI Day 3 (cmH_2_O/mmHg)	3.67 (2.03)	4.39 (2.82)	0.27

OI change from day 1 to day 3 (cmH_2_O/mmHg)	−3.32 (4.46)	−1.06 (1.97)	0.008

*FiO_2_: fraction inspired oxygen, mPaw: mean airway pressure, OI: oxygenation index, PaO_2_: arterial oxygen tension, PEEP: positive end expiratory pressure.

^#^Continuous variables were analyzed by Student's *t*-test or Mann-Whitney *U *test.

Variables are expressed as mean (standard deviation).

**Table 9:** Multivariate analysis of predictors of weaning failure in patients with severe acute respiratory failure.

Variables	Odds ratio	CI*	*P* values
Cerebrovascular accident	10.16	2.37–43.58	0.002
OI change from day 1 to day 3	0.74	0.51–1.08	0.12

*CI: confidence interval, OI: oxygenation index.
